# Calcium Signaling Modulator 4-MPTC Reduces Proliferative and Migration Activities of MDA-MB-468 Breast Cancer Cells

**DOI:** 10.1134/S0012496625600836

**Published:** 2026-03-30

**Authors:** A. Yu. Skopin, K. O. Gusev, D. A. Grekhnev, L. N. Glushankova, V. G. Kartsev, E. V. Kaznacheyeva

**Affiliations:** 1 Institute of Cytology, Russian Academy of Sciences, St. Petersburg, Russia; 2 InterBioScreen, Chernogolovka, Russia

**Keywords:** calcium, store-operated entry, proliferation, migration, breast cancer

## Abstract

Cell malignant transformation is accompanied by a remodeling of various cell life-support systems, including calcium signaling. Modulation of the calcium signal can significantly sensitize cancer cells, making them more sensitive to anticancer drugs. A search for selective compounds that influence the cell calcium response is a promising area in the development of combination therapies for oncology diseases. The aim of this study was to determine the effect of 4-MPTC on the invasive potential of triple-negative breast cancer cells. 4-MPTC was previously selected as a modulator of the Stim2-dependent calcium entry pathway via a small molecule screening. 4-MPTC was shown to induce a massive calcium release from the intracellular calcium store in the endoplasmic reticulum (ER), blocks store-operated calcium entry, and reduces proliferation and migration activity of MDA-MB-468 triple-negative breast cancer cells.

Studies have shown that store-operated calcium entry in cancer cells is often significantly higher than in untransformed cells. The observed deregulation of calcium signaling correlates with an increase in proliferation, invasion, and viability of cancer cells. A key role in store-operated calcium entry is played by the STIM proteins, which act as sensors of the calcium concentration in the endoplasmic reticulum (ER), and the Orai and TRPC proteins, which form ion channels in the plasma membrane. The functions of the proteins have been reviewed in detail [1]. Apart from affecting the cytosolic calcium concentration, an increase in the amplitude of store-operated calcium entry can facilitate an increase in calcium concentration in intracellular stores. Thus, to reduce the proliferative and migration potentials of cancer cells, it might be necessary not only to suppress store-operated calcium entry, but also to decrease the calcium concentration in the ER.

The STIM2 protein regulates the filling state of calcium stores and the basal calcium concentration in the cytoplasm in physiological conditions [2]. STIM2 protein overexpression has been detected in rectal and ER-positive breast cancers [3]. The role of STIM2 in pathological conditions is still poorly understood. A STIM2 knockdown has been shown to decrease motility of metastatic breast cancer cells by specifically inhibiting the NFAT1 signaling pathway, while an increase in STIM2 expression activates the NFAT1 signaling pathway and thus provokes metastasis, possibly by increasing the calcium concentration in the stores [4].

We have previously performed a screen of a substance library and selected 4-methyl-2-(2-propylpyridin-4-yl)-N-(pyridin-2-yl)thiazole-5-carboxamide (4-MPTC) as a modulator of STIM2-dependent calcium entry pathway (IC50 = 1 µM) [5]. The compound was further studied with the HEK293 cell line and MDA-MB-468 triple-negative breast cancer cells.

Changes in cytoplasmic calcium concentration in HEK293 cells were measured using the Fura-2AM fluorescent probe [6, 7]. 4-MPTC used to treat cells in a calcium-free solution caused a massive release of calcium from its intracellular stores (Fig. 1a). Subsequent treatment with 2 mM calcium led to extracellular calcium entry. 4-MPTC probably depletes the calcium stores in the ER or facilitates calcium release not only from the ER, but also from other organelles, such as mitochondria.

**Fig. 1.**
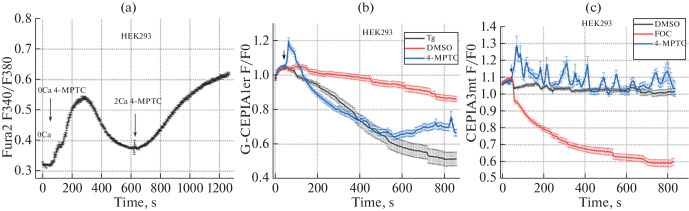
(a) Changes in calcium concentration in the cytoplasm of HEK293 cells incubated in a calcium-free medium in response to treatment with 100 µM 4-MPTC and 2 mM calcium. Plots show the time dependence of the ratio of Fura-2 fluorescence intensities upon excitation at 340 and 380 nm. Results are presented as the mean and standard error of the mean. (b) Effect of 4-MPTC on the calcium concentration in the ER in HEK293 cells. Plots show the time dependence of the G-CEPIA1er fluorescence intensity normalized to its initial value. The time point of supplementing the cell culture with 0.5% DMSO, 1 µM Tg, or 100 µM 4-MPTC is indicated with an arrow. Measurements were carried out in a calcium-free solution. (c) Effect of 4-MPTC on the calcium concentration in mitochondria in HEK293 cells. Plots show the time dependence of the CEPIA3mt fluorescence intensity normalized to its initial value. The time point of supplementing the cell culture with 0.5% DMSO, 1 µM Tg, or 100 µM 4-MPTC is indicated with an arrow. Measurements were carried out in a calcium-free solution.

Mitochondria and the ER were considered as the most likely calcium source sensitive to 4-MPTC. Changes in the calcium concentration in these organelles were measured using genetically encoded CEPIA3mt and G-CEPIA1er, which act as calcium sensors in mitochondria and the ER, respectively [8, 9]. These single-wavelength probes have an excitation wavelength of 470 nm and an emission wavelength of 510 nm. The higher the calcium concentration in organelles, the higher the fluorescence intensity of the sensors.

To study the effect of 4-MPTC on the ER calcium concentration, HEK293 cells were incubated in a calcium-free solution. As a control, we used 0.5% DMSO and 1 µM thapsigargin (Tg), which causes passive depletion of the ER calcium store.

Experiments with HEK293 cells were performed 24 h after their transfection with a plasmid coding for the G-CEPIA1er calcium sensor. The results of measurements are shown in Fig. 1b. Treatment with 100 µM 4-MPTC substantially reduced the calcium concentration in the ER as compared with the DMSO control and depleted the stores more intensely than Tg.

To study the effect of 4-MPTC on the calcium concentration in mitochondria, HEK293 cells were incubated in a calcium-free solution. As a control, we used 0.5% DMOS and a FOC solution, which contained three compounds: 3 µM FCCP, 3 µM oligomycin А, and 3 µM CGP-37157. FCCP acts as a protonophore and uncouples oxidative phosphorylation in mitochondria. FCCP is capable of depolarizing the mitochondrial membranes. Oligomycin A blocks oxidative phosphorylation and the electron transport chain by inhibiting mitochondrial membrane-associated ATP synthase. CGP-37157 acts as an inhibitor of the mitochondrial sodium/calcium exchanger. Acting together, the substances trigger a massive release of calcium from mitochondria.

Experiments with HEK293 cells were performed 24 h after their transfection with a plasmid coding for the CEPIA3mt calcium sensor. The results of measurements are shown in Fig. 1c. Treatment with 100 µM 4-MPTC caused oscillations of mitochondrial calcium concentration without decreasing the concentration relative to the DMSO control. The oscillations of mitochondrial calcium concentration probably arose because the cytoplasmic calcium concentration increased abruptly as a result of calcium release from the ER store.

Thus, we showed using the genetically encoded sensors that 4-MPTC substantially reduces the calcium concentration in the ER, but not in mitochondria.

An inhibitor analysis of 4-MPTC has been carried out previously with five breast cancer cell lines, and the MDA-MB-468 cell line has shown the greatest inhibition of the calcium response. The line was therefore chosen for further studies of the 4-MPTC effect on the calcium response phases in cancer cells. 4-MPTC caused depletion of Tg-sensitive ER calcium stores in MDA-MB-468 cells (Fig. 2b). To achieve maximum depletion of the intracellular calcium stores, we combined active calcium release induced through the IP_3_ receptor (treatment with 100 µM UTP) and blockage of calcium refilling of the stores (treatment with 1 µM Tg). When 4-MPTC was applied after maximum depletion of the stores (with Tg + UTP), the calcium concentration only slightly increased in the cytoplasm (Fig. 2a). Cell treatment with Tg + UTP after 4-MPTC treatment did not cause additional calcium release. Thus, 4-MPTC fully depleted the UTP-sensitive stores in MDA-MB-468 cells. In addition, 4-MPTC substantially (by 82 ± 2%) reduced store-operated calcium entry in these cells (Fig. 2c).

**Fig. 2.**
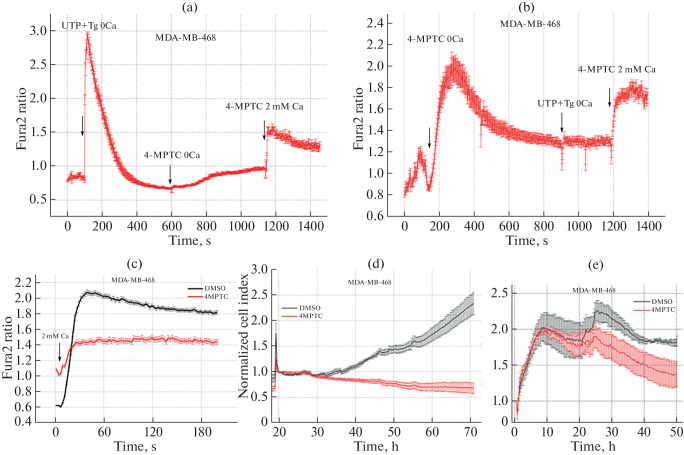
(a, b) Changes in calcium concentration in the cytoplasm of MDA-MB-468 cells incubated in a calcium-free solution in response to treatment with 100 µM 4-MPTC, 1 µM Tg, 100 µM UTP, and 2 mM Ca. 4-MPTC was added to the cells either after (a) or before (b) depletion of the calcium stores. (c) Store-operated calcium entry in the presence of 4-MPTC or DMSO (control); cells were treated for 10–15 min. Plots show the time dependence of the ratio of Fura-2 fluorescence intensities upon excitation at 340 and 380 nm. Results are presented as the mean and standard error of the mean. The time points of supplementing the cell culture with respective compounds are indicated with arrows. (d) Time dependence of the cell index, which reflects the amount of MDA-MB-468 cells. The culture medium was supplemented with 4-MPTC or DMSO 20 h after the start of the experiment. (e) Time dependence of the cell index, which reflects the amount of migrated MDA-MB-468 cells.

Literature data make it possible to assume that metastasis may depend not only on the amplitude of store-operated calcium entry, but also on the filling state of the ER calcium stores [10–12]. To verify the hypothesis, we studied how 4-MPTC affects migration and proliferative activities of MDA-MB-468 cells.

Migration and proliferative activities were evaluated using a xCELLigence real-time cell analyzer. The instrument measures the impedance between electrodes at the bottom of a special culture plate and calculates the cell index, which directly depends on the cell number and size [13]. Two variants of special plates are available for measurements with the instrument: one has single-chamber wells and is used for assessing proliferation (E-plate 16) and the other, two-chamber wells for assessing migration (CIM-plate 16). In the latter case, a nutrient concentration gradient is created between the chambers, and cells are thus forced to migrate from a chamber with a poor medium into a chamber with a rich medium, where the cell index is measured.

The effect of 4-MPTC on proliferation of MDA-MB-468 triple-negative breast cancer cells is shown in Fig. 2d. Compared with the DMSO control, 4-MPTC used at 10 µM for 48 h stopped cell proliferation and, moreover, exerted a cytotoxic effect. The cell index decreased by 30 ± 9% within 48 h.

Tests for migration were carried out using xCelligence with CIM-plate 16. Migration activity of MDA-MB-468 cells was found to decrease by 54 ± 20% after 48-h incubation with 4-MPTC (Fig. 2e).

Thus, 4-MPTC decreased proliferative and migration activities of breast cancer cells in addition to reducing the ER calcium concentration and suppressing calcium entry.
